# Effect of silkworm pupae (*Bombyx mori*) protein on colon cancer in nude mice: inhibition of tumor growth, oxidative stress and inflammatory response

**DOI:** 10.3389/fphar.2023.1138742

**Published:** 2023-07-19

**Authors:** Yaxi Zhou, Xiaojiao Ji, Diandian Wang, Yu Guo, Jian Zhao, Wenjie Yan

**Affiliations:** ^1^ College of Biochemical Engineering, Beijing Union University, Beijing, China; ^2^ Beijing Key Laboratory of Bioactive Substances and Functional Food, College of Biochemical Engineering, Beijing Union University, Beijing, China

**Keywords:** *Bombyx mori*, silkworm pupae protein, colon cancer, anti-tumor, inflammatory responses, health benefit

## Abstract

Silkworm pupa (*bombyx mori*) protein (SPP) is a potential therapeutic bioactive substance that has anti-tumor activity against breast, liver, and gastric cancers. The aim of this study was to investigate the antitumor effect of SPP on colon cancer nude mice. Using a subcutaneous tumor formation method, we validated the therapeutic effect of SPP on colon cancer nude mice *in vivo*. Results showed that SPP was cytotoxic to tumor cells. SPP could protect the liver of the nude mice by lowering hepatic oxidative stress and regulating serum inflammation levels by decreasing TNF-α and IL-2 levels while in-creasing INF-γ levels. In addition, diminished Ki-67 protein, enhanced cleaved caspase-3 protein, di-minished Vimentin, enhanced E-cadherin. These findings suggested that SPP’s antitumor activity may be achieved by reducing inflammation, inhibiting tumor proliferation and metastasis, and inducing apoptosis in cancer cells. In the future, SPP could be used as an anticancer drug, potentially providing a new source of drugs for the treatment of colon cancer.

## 1 Introduction

Cancer is a significant global public health issue in the 21st century (Siegel et al., 2022). In economically developed countries, cancer has emerged as a major disease that hampers life expectancy (Lin et al., 2021). Colon cancer, with approximately 10,861,000 annual deaths worldwide, ranks as the third most common cancer in men (Cronin et al., 2018). In China, colon cancer is also a leading cause of death and ranks third among the most prevalent malignant tumors, following lung and stomach cancers. Due to its high propensity to metastasize, colon cancer is among the deadliest cancers (Akouchekian et al., 2008; Tsilimigras et al., 2018).

Colon cancer treatment options, such as chemotherapy, surgery, and radiation therapy, can have potentially severe and toxic side effects (Kim et al., 2015). Consequently, there is an urgent need to develop new effective and non-toxic anti-tumor drugs. Numerous proteins with anti-tumor activity have been discovered in natural products, including phytohemagglutinin protein, ribosome-inactivating proteins, chlorogenic acid complex, and casein glycomacropeptide (Chen et al., 2013; Zeng et al., 2015; Gouthamchandra et al., 2017; Lagarda-Diaz et al., 2017). These proteins have demonstrated efficient and low-toxicity anti-tumor effects, making them promise therapeutic agents for cancer treatment. Additionally, studies have investigated the combined anti-tumor effects of various proteins or peptides with chemotherapeutic drugs (Wu et al., 2017; Reneeta et al., 2018). Bioactive proteins have advantages over anticancer drugs, as they do not accumulate toxicity and are less prone to drug resistance.

Edible insects are expected to reach a global market value of 80 billion by 2030 (Liceaga et al., 2022). Silkworm pupa protein (SPP), an insect protein, is not only nutritious but also economically valuable as a bioactive peptide with potential therapeutic effects (Zhou et al., 2022). Previous studies have demonstrated that SPP exhibits immune-enhancing, antibacterial, antioxidant, and anti-apoptotic properties (Guo et al., 2016; Baik and Rhee, 2017; Lee et al., 2017; Li et al., 2020). A recent study found that SPP can influence the energy metabolic process and promote apoptosis in colon cancer DLD-1 cells (Ji et al., 2022). A recent study found that SPP can affect the energy metabolic process and promote apoptosis in colon cancer DLD-1 cells.

In this study, we first established a subcutaneous tumorigenic model in nude mice using human colon cancer DLD-1 cells. Subsequently, we explored the anti-tumor effects of SPP in nude mice through *in vivo* experiments. Our focus was on the modulatory effects of SPP on oxidative stress, serum inflammation in the liver of tumor-bearing nude mice, and tumor inhibition. The objective of this study was to confirm the tumor-inhibitory effect of SPP and provide new potential therapeutic options for colon cancer. This research aims to establish a theoretical basis for further exploration and exploitation of SPP.

## 2 Materials and methods

### 2.1 Component analysis of SPP

SPP was purchased from Nantong Fuer Biological Products Co. An amino acid analyzer (Hitachi, Japan) was used to analyze the amino acid composition of SPP. The content of oligopeptides in SPP was determined by the Kjeldahl method. The molecular weight of SPP was identified by high performance liquid chromatography-electrospray ionization mass spectrometry (HPLC-ESI-MS). The mobile phase A contains 0.1% formic acid; the mobile phase B contains acetonitrile; the elution gradient is 0–50 min, 5%–95% B, 50–55 min, 95% B. Mass spectrometry conditions: electrospray ionization source (ESI) with positive ion mode scanning, sheath gas pressure of 35 arb, spray voltage of 4.5 kV, capillary temperature of 350°C, and capillary voltage of 35 V. The samples were first subjected to FT Full scan with resolution R set to 30,000 and secondary mass spectrometry with data dependent scan (DDS) in the scan range (m/z): 100–2000.

### 2.2 DLD-1 cell culture

Human colon cancer DLD-1 cells were purchased from the Cell Bank of the Chinese Academy of Sciences (catalog number TCHu134). After resuscitation, DLD-1 cells were cultured in RPMI-1640 medium (Genview, USA) containing 10% heat-inactivated fatal bovine serum (Gibco, USA) and 1% antibodies (100 U/mL penicillin, 100 mg/mL streptomycin) in a 37°C, 5% CO_2_ saturated humidity incubator (Zhou et al., 2018b).

### 2.3 Experimental animals

The protocols for the animal experiment were authorized by the Ethical Committee of Experimental Animal Care of Beijing Union University (approval number: 20200914) and were conducted following Chinese guidelines for animal welfare (GB/T35892–2018). Ninety female BALB/C nude mice were purchased from Beijing Huafukang Biotechnology Co. The nude mice were housed in separate cages in the SPF class animal room of the Health Food Function Testing Centre, College of Applied Arts and Sciences, Beijing Union University. The temperature was 26°C ± 1°C and the humidity was 60% ± 5%. The feed of nude mice were autoclaved, and the sterile bedding was changed once every 3–4 days.

### 2.4 Subcutaneous tumorigenic model of colon cancer cells in nude mice

Under aseptic conditions, DLD-1 cells in the logarithmic growth phase were digested with 0.25% trypsin, suspended in PBS, and prepared as a cell suspension under aseptic conditions, with the cell density adjusted to 2 × 10^6^ cells per 200 μL. Using the subcutaneous tumorigenesis method, DLD-1 cells were inoculated subcutaneously in nude mice, and each nude mouse was inoculated with 200 μL of cell suspension subcutaneously, and the subcutaneous tumorigenesis of nude mice was observed regularly.

### 2.5 Intervention setup

After 1 week of subcutaneous tumor injection, nude mice were randomly divided into 6 groups of 15 mice each. In total, six groups were setup as following. Control group, 75 mg/mL SPP group, 150 mg/mL SPP group, 300 mg/mL SPP group, 600 mg/mL SPP group, and 5-fluorouracil (5-FU, purchased from Wuhan Saiwei Biotechnology Co., Ltd., Wuhan, China) group. Among them, the SPP group and 5-FU group received oral gavage administration. The mice were weighed every other day after each administration, and the tumor volume was measured once every 4 days with vernier calipers. Twenty-four hours after the last dose, body weight was recorded, and mice were executed by the cervical dislocation method after blood sampling from the orbits, and tumor masses, liver, colon, rectum, and spleen tissues were dissected and peeled out. The stripped tumor masses were measured and weighed, and photographs were taken. The tumors were divided into two parts, one soaked in 4% paraformaldehyde and the other stored in the refrigerator at −80°C for backup; the spleen was weighed; the colon and rectum were soaked in 10% formalin.

### 2.6 Calculation of tumor volume, tumor suppression rate, and organ index

Tumor volume in nude mice = 0.5 × tumor length (mm) × tumor width (mm)^2^ (Tomayko and Reynolds, 1989).

Tumor suppression rate (%) = (average tumor weight of control group - average tumor weight of drug administration group)/average tumor weight of control group × 100% (Stribbling and Ryan, 2022).

Visceral index (%) = Visceral weight/body weight × 100%.

### 2.7 Hematoxylin-eosin staining for histopathological changes

Tumors, colon, and rectum (embedded in paraffin wax) were cut into 8-µm-thick sections with a slicer, soaked in xylene for 10 min for dewaxing, and soaked in anhydrous ethanol for 5 min to elute the xylene. The slices were sequentially placed in 95%, 85%, and 70% ethanol for 5 min each for hydration. The tissue sections were stained with hematoxylin for 10 min and eosin for 3 min at room temperature. After staining, the tissue sections were subjected to gradient dehydration (80% ethanol for 5 s, 95% ethanol for 2 min, and anhydrous ethanol for 2 min). The pathological changes of each tissue were observed under the light microscope after air-drying and photographed.

### 2.8 Liver antioxidant index assay

The liver was accurately weighed and added to 9 times the volume of 0.9% saline at a ratio of weight (g) to volume (mL) = 1:9, and the homogenate was mechanically homogenized under ice-water bath conditions to prepare a 10% homogenate, centrifuged at 2,500–3,000 rpm for 10 min. The supernatant was taken and diluted with saline, and the following five indexes were determined by the kit method: malondialdehyde (MDA), superoxide (SOD), catalase (CAT), glutathione aminotransferase (GPT), and glutamic oxaloacetic aminotransferase (GOT). All kits were purchased from Nanjing Jiancheng Technology Co., Ltd. For each sample we performed 3 replicate measurements and took the average.

### 2.9 ELISA assay for the determination of inflammatory cytokines in serum

Whole blood from nude mice was centrifuged at 3,000 rpm for 15 min in a centrifuge at 4°C, and the supernatant was taken for immediate detection. Tumor necrosis factor -α (TNF-α), Interleukin-2 (IL-2), and Interferon-γ (INF-γ) ELISA kits were chosen according to the instructions. ELISA kits were purchased from Wuhan Huamei Biological Engineering Co., Ltd. Three technical replicates were performed for each pool in each group, the standard curves were plotted, and the assay results were expressed as optical density (O.D.).

### 2.10 Immunohistochemical staining detection

After dewaxing, antigen repair, and sealing of tumor tissue sections, antibodies were added according to the instructions of the immunohistochemistry kit, followed by DAB color development, hematoxylin re-staining, and neutral resin sealing of the sections. Vimentin, E-cadherin, Ki-67, cleaved caspase-3 (C-caspase-3) antibodies were purchased from Wuhan Xavier Biotechnology Co., Ltd. Wuhan, China. Five representative fields of view of each tissue section were observed randomly under the light microscope. The mean optical density (mean IOD) was calculated for each slice using Image Pro Plus 6.0 image analysis software.

### 2.11 Statistical analysis

The final data were presented as Mean ± S.D. Experimental differences were assessed by one-way ANOVA using SPSS version 24.0 (IBM). Differences among more than two groups were analyzed by one-way ANOVA following Tukey’s test.

## 3 Results

### 3.1 Analysis results of SPP composition

Table 1 demonstrates the amino acid composition of SPP. Among them, the highest content of glutamic acid was 11.4 g/100 g, and the lowest content of histidine was 1.87 g/100 g. The amino acids of SPP were complete, and the essential amino acids accounted for 62.5% of the non-essential amino acid content and 38.4% of the total amino acid content. It indicates that the amino acid composition of SPP is an ideal amino acid composition pattern and fully conforms to the standard pattern recommended by FAO/WHO. The oligopeptide has a strong active function (Ji et al., 2017). This SPP oligopeptide’s content is 77.5 g/100 g.

**TABLE 1:** Temperature and wildlife count in the three areas covered by the study.

Amino acid categories	Content (g/100 g)	Amino acid categories	Content (g/100 g)
Aspartic acid	10.2	tyrosine	4.64
Threonine	4.10	Phenylalanine	3.67
serine	3.80	Lysine	5.69
glutamic acid	11.40	Histidine	1.87
Glycine	3.38	Arginine	4.35
Alanine	4.18	Proline	3.84
Valine	4.63	Total amino acids	77.50
Methionine	2.37	Essential amino acids/non-essential amino acids	0.625
Isoleucine	3.61	Essential amino acids/amino acid total	0.384
Leucine	5.73	Oligopeptides	75.00

**FIGURE 1:**
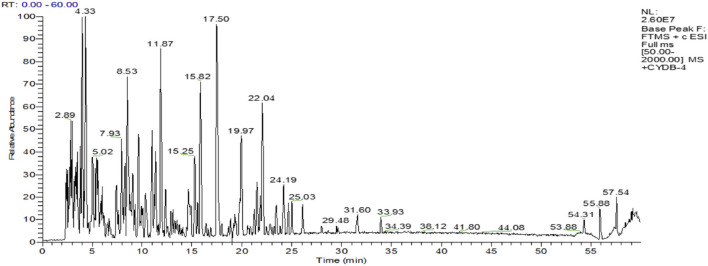
The total ion flow diagram of SPP.

HPLC-ESI-MS has both the high separation performance of liquid chromatography and the high sensitivity and detection ability of electrospray ionization mass spectrometry. We measured the molecular weight range of SPP by HPLC-ESI-MS. The findings revealed that the molecular weight range of SPP was less than 500, with the majority concentrated in the 200–400 range. Figure 1 shows the total ion flow diagram of SPP.

### 3.2 SPP inhibits colon cancer tumor growth in nude mice *in vivo* but does not affect normal growth in model nude mice

**FIGURE 2:**
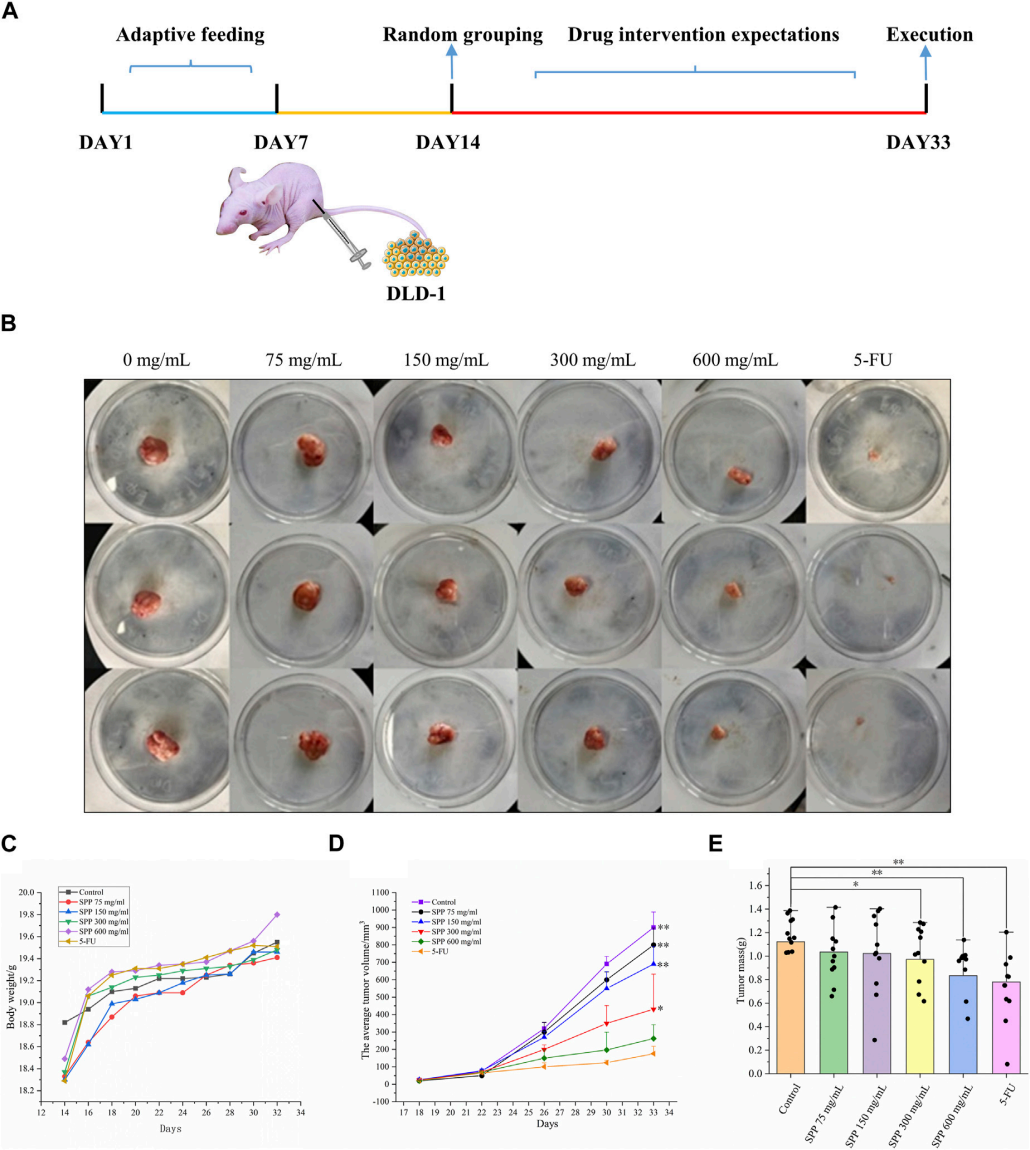
The impact of various experimental groups on the body weight and tumor volume of DLD-1 tumor-bearing mice. **(A)** Schematic diagram of the experimental procedure; **(B)** Tumor image in nude mice; **(C)** Trend of average body weight change; **(D)** Tumor volume change; **(E)** Average tumor weight (*n* = 14). Values are means ± S.D., *p < 0.05 vs. control group, **p < 0.01 vs. control group.

In this experiment, it was clearly observed that the tumor volumes in the SPP-treated group and the positive control 5-FU group were smaller than those in the control group after the end of the administration. According to the statistics of each vernier caliper measurement, after the 4th gavage of SPP, the growth rate of tumor volume in the SPP-treated group slowed down compared with that in the control group, and after the 10th sample injection, the difference in tumor volume in each treated group compared with that in the control group became extremely obvious. After the nude mice were executed, the tumors were measured with calipers. The results showed that the tumor increments in the SPP-treated (780 mm^3^, 664 mm^3^, 407 mm^3^, 243 mm^3^) and 5-FU groups (153 mm^3^) were lower than those in the control group (875 mm^3^), and there was a highly significant difference (*p < 0.01*). This statement demonstrated that SPP significantly inhibited the growth and proliferation of DLD-1 colon cancer tumors (Figures 2B, D, E). Table 2 presents the tumor volume data obtained for each group for each measurement. Interestingly, there was no difference in the weight gain of the animals in each group before and after the test, but the analysis of the specific data from the weighing showed that the trend of the average weight gain of the animals in each group was significantly different. Compared with the control group, the 600 mg/mL group showed a sharp trend of mean body weight gain in the early period and a slow increase in the middle. The rapid increase at the beginning was probably caused by the rapid proliferation of tumors, resulting in increased feeding of the animals, and the weight gain slowed down at the later stage when the tumor growth entered a plateau (Figure 2C; Table 3). The tumor suppression rate was calculated according to the formula. We found that the mean tumor weight of SPP-treated groups was all smaller than that of the control group, with significant differences in the 300 mg/mL group (*p < 0.05*), highly significant differences in the 600 mg/mL group (*p < 0.01*), and highly significant differences in the 5-FU group compared with the control group (*p < 0.01*). Among the SPP-treated groups, the 600 mg/mL group had the highest tumor suppression rate of 36.19%. In addition, compared with the positive control 5-FU group, there was no difference in the tumor suppression effect between the 600 mg/mL group and 5-FU, indicating the significant tumor suppression effect of SPP on DLD-1 tumor-bearing nude mice (Table 4).

**TABLE 2:** tumour volume data obtained from each group for each measurement.

Times	Control (mm^3^)	SPP 75 mg/mL (mm^3^)	SPP 150 mg/mL (mm^3^)	SPP 300 mg/mL (mm^3^)	SPP 600 mg/mL (mm^3^)	5-FU (mm^3^)
1	25 ± 0.2	20 ± 1.1	26 ± 2.1	24 ± 3.3	20 ± 0.2	23 ± 1.2
2	75 ± 5.3	50 ± 2.7	78 ± 10	70 ± 8.7	69 ± 0.9	65 ± 7.8
3	320 ± 34	300 ± 56	270 ± 34	200 ± 26	150 ± 47	100 ± 23
4	691 ± 43	600 ± 45	550 ± 97	350 ± 102	197 ± 1.2	124 ± 13
5	900 ± 89	800 ± 97	690 ± 102*	431 ± 201**	263 ± 79**	176 ± 42**

Tumor volume was measured every 4 days, *n* = 15.

*p < 0.05 vs. control group.

**p < 0.01 vs. control group.

**TABLE 3:** Effect of SPP-treatment on the weight of nude mice.

Number of gavages	Control (g)	SPP 75 mg/mL(g)	SPP 150 mg/mL(g)	SPP 300 mg/mL(g)	SPP 600 mg/mL(g)	5-FU (g)
1	18.33 ± 1.14	18.82 ± 1.51	18.30 ± 1.81	18.37 ± 0.88	18.49 ± 1.22	18.29 ± 1.45
2	18.84 ± 0.92	18.94 ± 1.10	18.62 ± 1.35	19.06 ± 0.64	19.12 ± 0.83	19.06 ± 0.99
3	18.97 ± 0.85	19.10 ± 0.90	18.99 ± 1.38	19.14 ± 0.89	19.28 ± 0.82	19.25 ± 0.78
4	19.06 ± 0.90	19.13 ± 0.82	19.03 ± 1.50	19.23 ± 0.76	19.29 ± 0.85	19.31 ± 0.91
5	19.09 ± 0.87	19.22 ± 0.69	19.09 ± 1.15	19.25 ± 0.79	19.34 ± 0.81	19.31 ± 0.83
6	19.09 ± 0.92	19.22 ± 0.66	19.18 ± 0.80	19.29 ± 0.81	19.35 ± 0.76	19.35 ± 1.01
7	19.25 ± 0.98	19.23 ± 0.84	19.25 ± 0.88	19.31 ± 0.68	19.37 ± 0.83	19.41 ± 0.86
8	19.34 ± 1.00	19.26 ± 1.33	19.26 ± 1.06	19.33 ± 0.71	19.47 ± 0.77	19.47 ± 0.89
9	19.36 ± 0.88	19.45 ± 0.44	19.46 ± 1.51	19.39 ± 0.79	19.56 ± 1.21	19.52 ± 0.71
10	19.41 ± 0.80	19.55 ± 0.64	19.46 ± 1.10	19.48 ± 0.87	19.80 ± 2.25	19.51 ± 1.20

Nude mice were administered every 2 days and weighed every other day after administration, *n* = 15.

**TABLE 4:** Tumor suppressive effect of each treatment group on tumor-bearing mice.

Group	Dosage	Tumor mass/g	Tumor suppression rate/%
control	—	1.12 ± 0.18	—
SPP	75 mg/mL	1.03 ± 0.23	8.04
	150 mg/mL	1.02 ± 0.31	8.93
	300 mg/mL	0.97 ± 0.29*	13.4
	600 mg/mL	0.83 ± 0.26**	25.89
5-FU	20 mg/kg	0.78 ± 0.30**	30.36

Tumors were removed anatomically for weighing, and the tumor suppression rate was calculated. Values are means ± S.D., *n* = 14*.*

*p < 0.05 vs. control group.

**p < 0.01 vs. control group.

According to Table 5, it can be concluded that the spleen coefficients of all treatment groups decreased to different degrees compared with the control group, with the 600 mg/mL group showing the greatest decrease in spleen coefficients compared the control group. The liver coefficients of all treatment groups were smaller than those of the control group, and the 5-FU group showed the greatest decrease compared with the control group, followed by the 600 mg/mL group. However, there was no significant difference between the spleen index and liver index in different groups (*p* > 0.05). This also indicates that SPP does not affect the normal growth of nude mice. SPP is cytotoxic to tumor cells and does not damage other tissues in nude mice.

**TABLE 5:** Effect of each treatment group on the organ index of tumor-bearing mice.

Group	Dosage	Spleen index/%	Liver index/%
Control	—	0.3495 ± 0.0014	5.10 ± 0.27
SPP	75 mg/mL	0.2797 ± 0.0010	5.06 ± 0.32
	150 mg/mL	0.2862 ± 0.0012	5.00 ± 0.36
	300 mg/mL	0.2845 ± 0.0007	5.08 ± 0.33
	600 mg/mL	0.2429 ± 0.0004	4.92 ± 0.34
5-FU	20 mg/kg	0.2533 ± 0.0007	4.72 ± 0.28

The spleen and liver were removed by dissection, weighed, and the organ index was calculated. Values are means ± S.D., n = 15.

**FIGURE 3:**
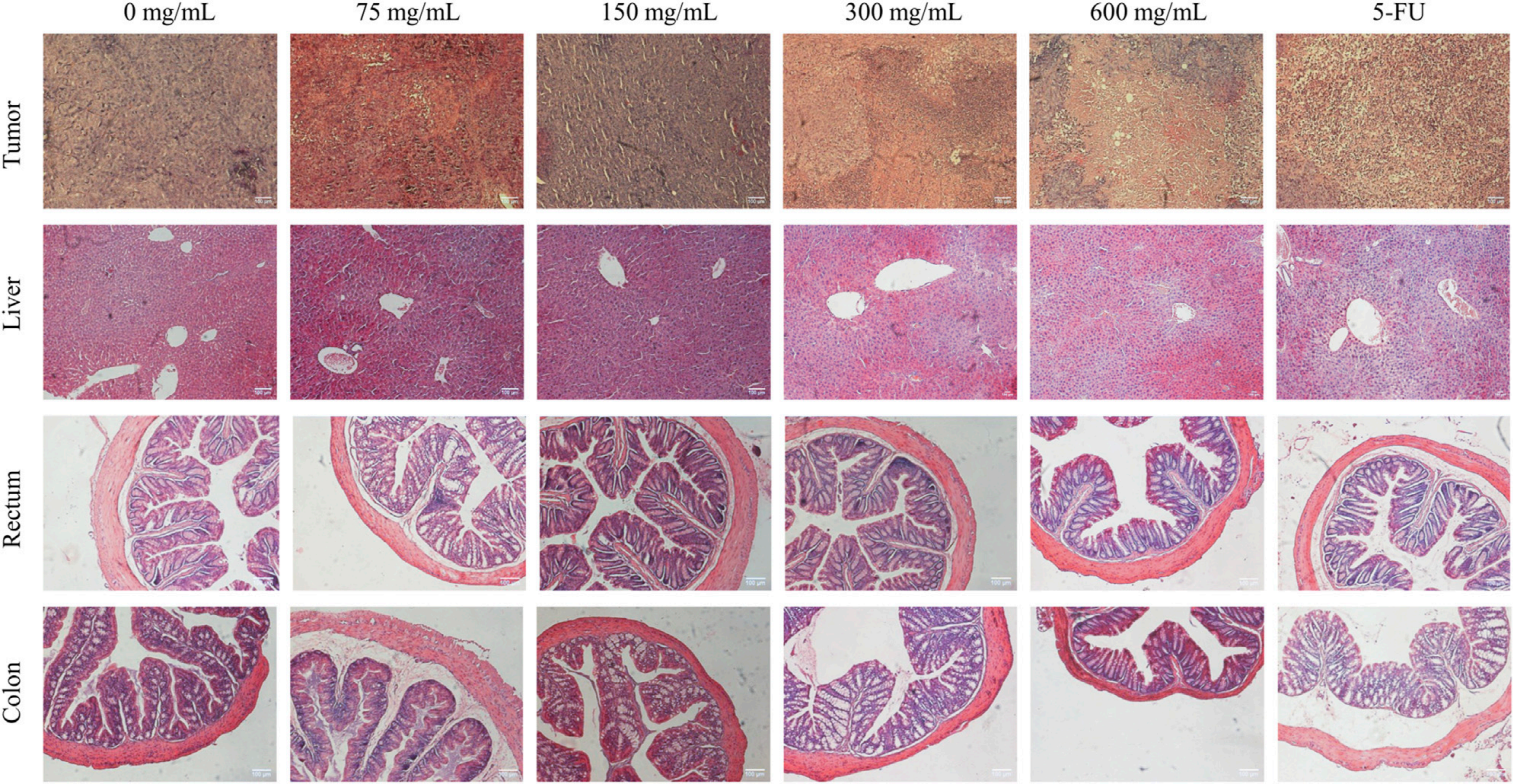
Effect of SPP on histopathological changes in tumor, liver, colon, and rectal tissues of DLD-1-loaded human colon cancer mice.

The tumor HE staining results showed that the tumor cells in the control group were growing vigorously, the nuclei were large and deeply stained, the tumor cells were irregularly arranged, the growth distribution was uniform and close, and there were a large number of capillaries in the interstitial space of the tumor tissues with the deep distribution. With the increase in SPP concentration, the distribution of tumor cells became more and more sparse, the degree of vacuolation increased, and the tumor cells appeared necrotic. The nucleus appeared ruptured and even lysed, and the structure was unclear. The tissues were filled with red blood cells and there was an obvious bleeding phenomenon. This indicates that SPP is cytotoxic to tumor cells (Figure 3).

The liver HE staining results showed that the liver of all groups of SPP mice had normal morphology, intact hepatocytes, and complete hepatic lobules, and there were no pathological changes of toxic damage compared with the control group, indicating that SPP had no toxic damage effect on the liver. We hypothesized that the side effects of the chemotherapeutic drug 5-FU were the cause. Rectum HE staining showed no significant changes in muscle thickness and mucosal thickness in the SPP group compared with the control group, and the intestinal wall was thinner in the 5-FU group compared with the other groups. The HE staining of the colon showed that there were no significant changes in muscle layer thickness and mucosal thickness in the 75 mg/mL, 150 mg/mL, and 300 mg/mL groups compared with the control group, and the intestinal wall was thinner and the villi were shorter in the 600 mg/mL and 5-FU groups. Suggesting that SPP has no damaging effect on the colon and rectum (Figure 3).

### 3.3 SPP alleviates the level of oxidative stress in the liver of tumor-bearing mice

**FIGURE 4:**
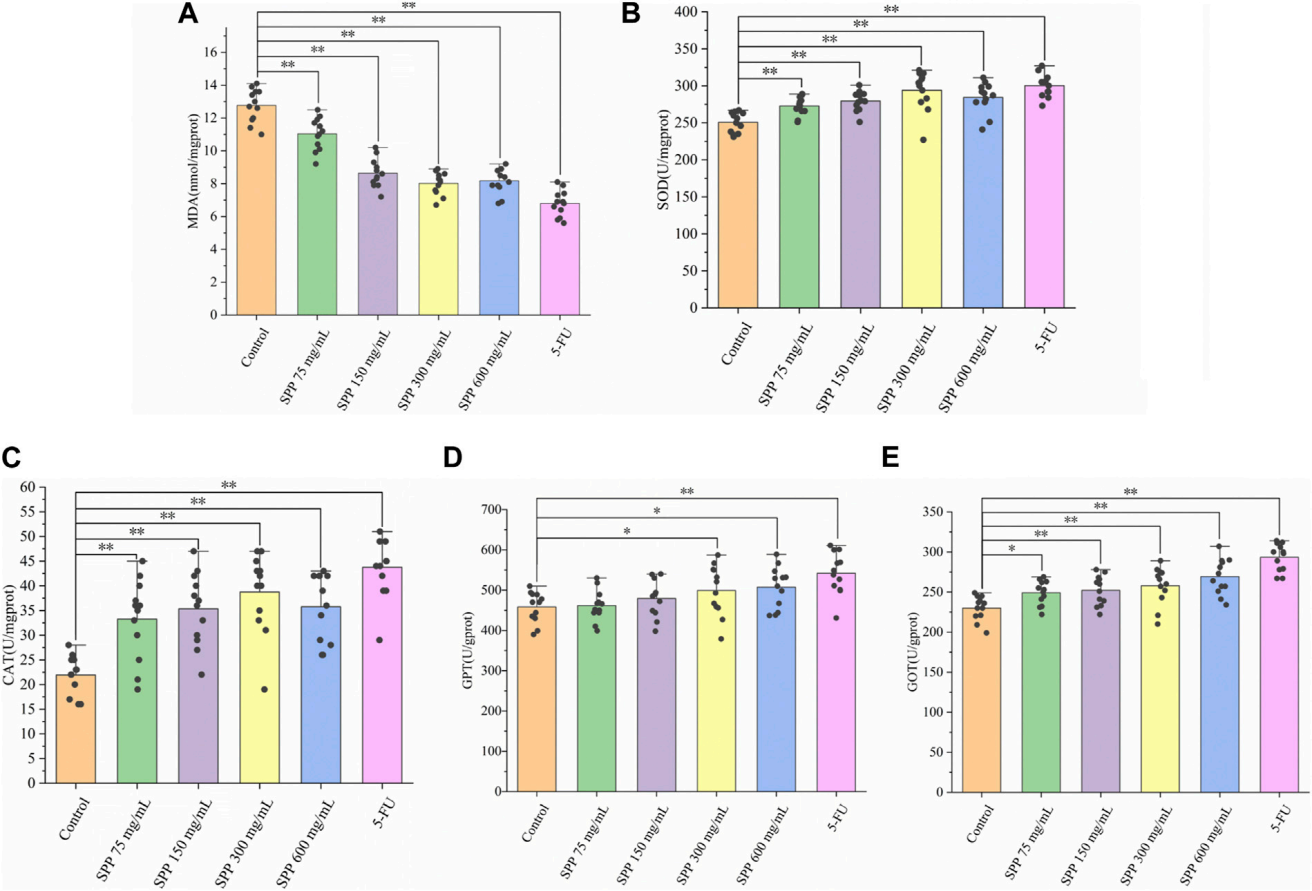
The impact of SPP on the oxidative-related indicators in the liver of nude mice. **(A)** MDA; **(B)** MOD; **(C)** CAT; **(D)** GPT; **(E)** GOT. Values are means ± S.D., *n* = 12. *p < 0.05 vs. control group, **p < 0.01 vs. control group.

MDA levels usually respond to the degree of lipid peroxidation in the body and indirectly to the degree of oxidative damage in the liver. The level of SOD activity indirectly responds to the ability of the body to scavenge oxygen free radicals. The level of MDA in turn indirectly reflects the severity of the free radical attack on body cells. MDA is often detected in conjunction with SOD (Marjaneh et al., 2018). The liver MDA levels were significantly lower in the SPP-treated and 5-FU groups compared to the control group, and the difference was highly significant (*p < 0.01*). The tumor suppressive effect was shown to reduce liver MDA levels to some extent and was correlated with the tumor suppression rate (Figure 4A). Compared with the liver SOD activity in the control group, the SPP-treated group and the 5-FU group both showed different degrees of enhancement, and the difference was highly significant (*p < 0.01*) (Figure 4B).

CAT is an important antioxidant enzyme in the process of biological oxidation, effectively scavenging various reactive oxygen groups and thus preventing damage to the cell membrane system by these groups. Changes in CAT activity can accurately reflect whether external environmental conditions are stressing the cell for survival. Compared with the liver CAT activity in the control group, there was a different degree of increase in the SPP-treated and 5-FU groups, with significant differences (*p < 0.01*) (Figure 4C). Liver GPT activity was significantly increased in all treatment groups compared with the control group, with significant differences in the 75 mg/mL group (*p < 0.05*) and highly significant differences in all other groups (*p < 0.01*) (Figure 4D). Compared with the control group, the liver GOT level increased to different degrees in all treatment groups, and the differences were highly significant (*p < 0.01*) (Figure 4E). The transaminase indexes showed that the 600 mg/mL group in the SPP dose group had the strongest degree of effect on hepatic GOT and GPT activities. In addition, in all three indices of CAT, GPT, and GOT, the SPP dose group and 5-FU group showed highly significant differences compared with the control group (*p < 0.01*). This could indicate that the effect of SPP on the liver of nude mice is similar to that of the chemotherapeutic agent 5-FU.

### 3.4 SPP downregulated pro-inflammatory factors TNF-α and IL-2, and upregulated INF-γ levels

**FIGURE 5:**
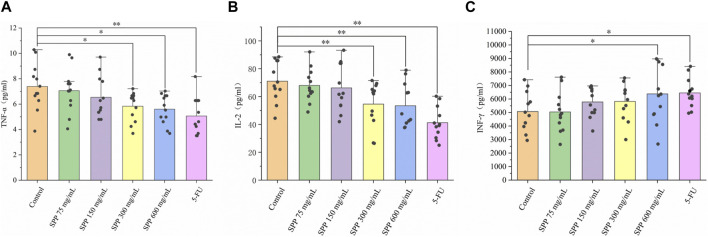
The impact of SPP on the inflammation-related indicators in the liver of nude mice. **(A)** TNF-α; **(B)** IL-2; **(C)** INF-γ. Values are means ± S.D., *n* = 12. *p < 0.05 vs. control group, **p < 0.01 vs. control group.

We used an enzyme-linked immunosorbent assay (ELISA) to detect TNF-α, IL-2, and INF-γ extracellular proteins in the serum of nude mice, and this method can detect cytokines at the protein level. TNF-α levels were lower in the SPP dose and 5-FU groups compared to the control group, with the SPP 600 mg/mL-treated group showing the greatest reduction (Figure 5A). The levels of IL-2 and INF-γ in the SPP dose group were significantly lower and higher, respectively than in the control group (*p < 0.01*), with a concentration gradient (Figures 5B, C). In addition, the inhibitory effect of SPP on pro-inflammatory cytokines was dose-dependently increased. And, the effects of 600 mg/mL SPP and the positive drug 5-FU groups on cytokines in serum were comparable.

### 3.5 SPP inhibits tumor cell proliferation, induces apoptosis and suppresses colon cancer tumor metastasis in nude mice

**FIGURE 6:**
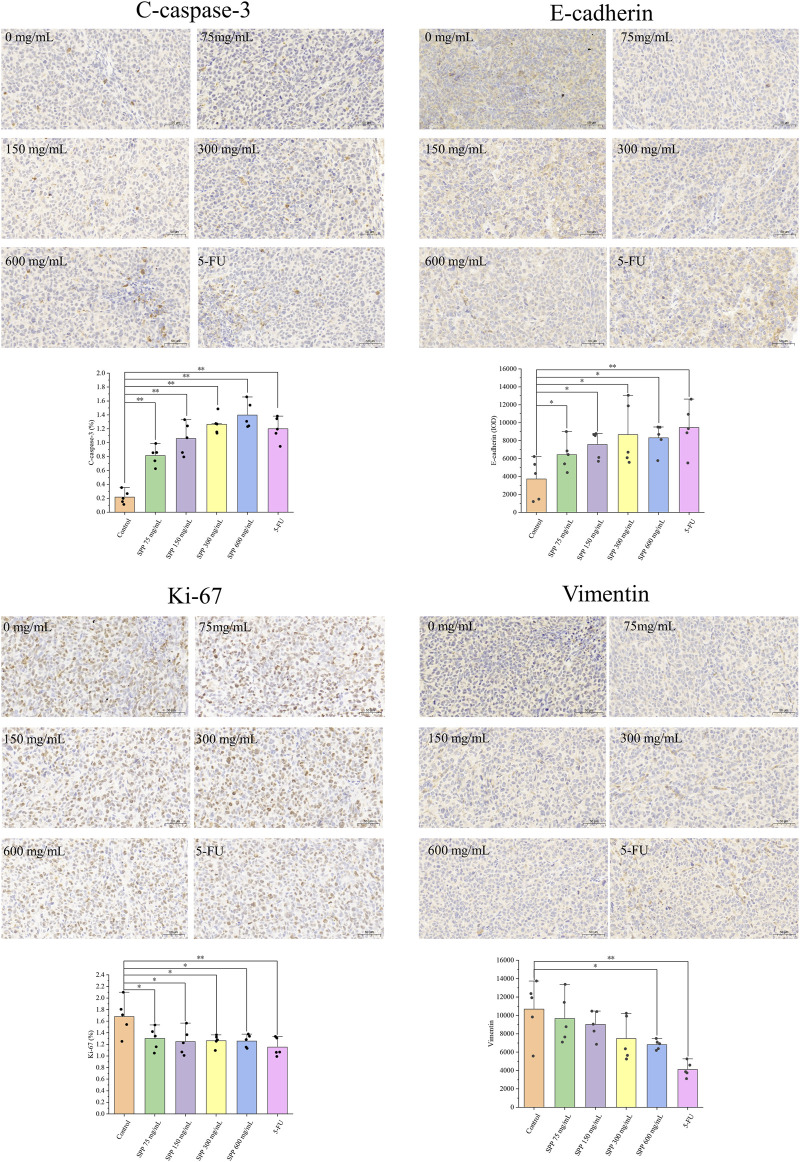
Immunohistochemical detection of the effect of each treatment group on tumor tissue protein expression. C-caspase-3, Ki-67 were calculated using ImageJ software for the percentage of positive cells. Vimentin, E-cadherin were calculated as mean optical density values using Image Pro Plus 6.0 software. *n* = 5. *p < 0.05 vs. control group, **p < 0.01 vs. control group.

The effect of SPP treatment on the expression of proliferation, apoptosis, and migration-related proteins in tumor tissues was examined by immunohistochemical staining. Vimentin, which maintains the mesothelial phenotype, showed weaker staining compared with the control group, and E-cadherin, which maintains the epithelial phenotype, showed enhanced staining. In addition, Ki-67 protein expression was reduced, and C-caspase-3 protein expression was enhanced (Figure 6). Based on these results, it can be tentatively concluded that SPP inhibited the growth of colon cancer tumors in nude mice by inhibiting tumor cell proliferation and inducing tumor cell apoptosis. And it could inhibit the metastasis of colon cancer tumors.

## 4 Discussion

Silkworm pupae have excellent health properties and have been found to lower blood pressure and blood lipids (Ryu, 2014) and also reduce the risk of Alzheimer’s disease (Wattanathorn et al., 2012). Current studies have demonstrated that silkworm pupae protein can inhibit the inflammatory response triggered by human breast cancer (Chukiatsiri et al., 2020) and also inhibit the growth of gastric cancer SGC-7901 cells through the apoptotic pathway (Weixin et al., 2019). In addition, the selenium-rich amino acids in silkworm pupae protein can induce apoptosis in human hepatocellular carcinoma cells SMMC-7721 and achieve hepatocarcinoma inhibition (Hu et al., 2005). These studies confirmed the antitumor activity of silkworm pupae protein.

As a chemotherapeutic agent, 5-FU has anti-tumor cell metabolic effects in the colon, rectal, and breast cancers, and is a good chemotherapeutic agent that is widely used in the treatment of solid malignancies (Capitain et al., 2008). However, 5-FU has side effects when used in chemotherapy. In patients with a deficiency of dihydropyridine dehydrogenase (DPD) activity, symptoms of 5-FU intolerance can occur (Kaldate et al., 2012). Moreover, 5-FU is not effective in the treatment of patients with advanced colon cancer, with high resistance and low response rates (Amerizadeh et al., 2018).

The results of HE staining showed that the liver of the positive control 5-FU group showed obvious cell breakage and ablation, and the thickness of the intestinal tissue muscle layer was reduced, the intestinal villi were shortened, and the mucosa was thinner, indicating that 5-FU as a chemotherapeutic agent for tumor treatment can cause a certain degree of toxicity and killing effect on the organs and tissues of the body, and even functional damage and disorders may occur. Therefore, to reduce the side effects of chemotherapy, it is necessary to use natural compounds as alternative therapeutic agents to 5-FU. Through *in vivo* experiments in nude mice, we investigated the antitumor activity of SPP from silkworm pupae against human colon cancer DLD-1 tumor-bearing nude mice. From the experimental results, we can see that SPP does not affect the normal growth of nude mice and has no toxic effect on nude mice. However, SPP has inhibitory and toxic effects on tumor tissues and tumor cells. This is the advantage of the anti-tumor effect of SPP, which reduces or even eliminates the damage caused by the chemotherapeutic drug 5-FU to the organism. The combination of SPP and 5-FU in cancer treatment has been reported to reduce the toxic effects of 5-FU on healthy tissues (Reneeta et al., 2018).

Oxidative stress is associated with several cancers (Hayes et al., 2020). In general, cancer patients have mostly low levels of antioxidant enzymes and high levels of MDA. The correlated levels of these biomarkers are closely related to disease progression (Jelic et al., 2021). In our experiment, we evaluated malondialdehyde (MDA), superoxide dismutase (SOD), and catalase (CAT) levels in the liver to examine oxidative stress. From the results, we can see that MDA was significantly increased and SOD and CAT were significantly decreased in the livers of nude mice in the control. Increased MDA indicates increased levels of oxidative stress and decreased levels of antioxidant defense (Peddireddy et al., 2012). Decreased SOD and CAT lead to impairment of antioxidant defense mechanisms, which in turn can lead to oxidative stress. Compared to the control group, MDA levels were significantly decreased, and SOD and CAT were significantly increased in the liver of nude mice after SPP intervention. This is similar to other findings in colon cancer (Gill et al., 2016), indicating that SPP ameliorates oxidative stress in nude mice. GPT and GOT are biomarkers that respond to liver injury (Martinello et al., 2017), and the levels of both indicators are proportional to the degree of hepatocyte damage. Compared to the control group, the content of GPT and GOT increased, which indicated that the liver of nude mice was damaged. The GPT and GOT content were significantly lower compared to the 5-FU group. This indicates that SPP does not inhibit tumors by protecting the liver, but it reduces liver damage in nude mice compared with the chemotherapeutic drug 5-FU.

The inflammatory response is closely linked to tumorigenesis. Chronic inflammation is involved in various steps of tumorigenesis, including cell transformation, survival, proliferation, invasion, angiogenesis, and metastasis (Hirano et al., 2020). And, the ability of inflammation to induce colon cancer has been reported (Okada et al., 2000). Altered levels of pro-inflammatory and anti-inflammatory cytokines in cancer demonstrate a strong correlation between inflammation and cancer (Kaur et al., 2018). We predicted that inflammatory responses would also occur *in vivo* in tumor-bearing nude mice. SPP reduced TNF-α and IL-2 levels while increasing INF-γ levels in the serum of tumor-bearing nude mice (Figure 4). TNF-α is an inflammatory factor that plays a role in the systemic inflammatory response and can induce apoptosis (Li et al., 2010). TNF-α is also used to treat cancer (Szlosarek et al., 2006; Grimm et al., 2011). TNF-α has been found to play an important role in colon cancer research (Sakai et al., 2010; Singh et al., 2020).

Similarly, IL-2 has been associated with the progression of colon cancer (Berghella et al., 1994). SPP significantly reduced TNF-α and IL-2 in tumor-bearing nude mice in our experiments, implying that SPP improves the serum inflammatory response in nude mice. INF-γ is a cytokine with anti-tumor activity that promotes pathological inflammatory processes in the tumor microenvironment (Alspach et al., 2019; Gocher et al., 2021). In our results, the level of INF-γ protein expression was significantly increased in the SPP-treated group. We hypothesize that SPP reduces the inflammatory response in nude mouse serum by decreasing TNF-α and IL-2 levels while increasing INF-γ levels.

Furthermore, we examined the expression of Ki-67, C-caspase-3, Vimentin, and E-cadherin proteins. The results showed that the expression of Ki-67, a cell proliferation marker protein, was significantly decreased; the expression of apoptosis-inducing shear C-caspase-3 was significantly increased, indicating that SPP also exerted significant anti-tumor effects in nude mice. Ki-67 is a proliferation marker of human tumor cells (Menon et al., 2019), and Ki-67 staining was diminished, indicating that the proliferation of cancer tissue cells is resistant to resistance (Wang et al., 2020). C-caspase-3 can cause apoptosis in tumor cells and has been used by many researchers as a surrogate marker for the efficacy of cancer therapy (Galluzzi et al., 2016). It has been shown that C-caspase-3 can regulate the migration, invasion, and metastasis of colon cancer cells (Zhou et al., 2018a). Therefore, we speculate that the antitumor effect of SPP is achieved by inhibiting the proliferation of DLD-1 cells and inducing their apoptosis. Vimentin and E-cadherin proteins serve as cell migration-related markers, and the expression of these two proteins tends to be inversely correlated. Their expression can reflect whether SPP has anti-colon cancer metastatic potential *in vivo* (Han et al., 2020; Niknami et al., 2020).

Our results showed that Vimentin staining was attenuated, and E-cadherin staining was enhanced compared to the control group. E-cadherin inactivation drives colon cancer metastasis (Alberti et al., 2021). Apparently, SPP promotes E-cadherin expression, and therefore, we confirmed the ability of SPP to inhibit the metastasis of human DLD-1 colon cancer cells in nude mice. In conclusion, SPP can play a role in inhibiting colon cancer tumor cell proliferation, inducing apoptosis, and suppressing tumor metastasis in nude mice.

## 5 Conclusion

Our results suggest that SPP is cytotoxic to human DLD-1 colon cancer cells and has a potential antitumor effect on the human DLD-1 colon cancer model. This may be mediated by inhibiting tumor cell proliferation and promoting tumor cell apoptosis. In addition, SPP ameliorated oxidative stress and serum inflammatory responses in the liver of hormonal nude mice and was able to prevent tumor metastasis in nude mice. These results may provide a new option for the treatment of colon cancer. However, this study did not thoroughly investigate its mechanism of action, and the structure of SPP has not been clearly characterized. In subsequent research, we will conduct structural characterization of SPP and further explore its potential pathways for anti-colorectal cancer effects.

## Data Availability

The original contributions presented in the study are included in the article/supplementary material, further inquiries can be directed to the corresponding author.
